# Monitored Anesthesia Care and Soft-Tissue Infiltration With Local Anesthesia for Short Cephalomedullary Nailing in Medically Complex Patients: A Technique Guide

**DOI:** 10.7759/cureus.20624

**Published:** 2021-12-22

**Authors:** Andrew S Bi, Nina D Fisher, Abhishek Ganta, Sanjit R Konda

**Affiliations:** 1 Orthopedic Surgery, NYU Langone Health, New York, USA

**Keywords:** hip fracture, mac-stila, monitored anesthesia care, local anesthesia, short cephalomedullary nailing

## Abstract

Hip fractures are increasingly common and often occur in patients with complex medical comorbidities. There remains a need for a safer anesthetic option for these patients for the operative repair of their injury other than general or neuraxial anesthesia. At our institution, for medically complex and physiologically tenuous patients, we perform Monitored Anesthesia Care and Soft-Tissue Infiltration of Local Anesthetic (MAC-STILA) when performing percutaneous fixation techniques for hip fractures. We describe our technique here.

## Introduction

Hip fractures are increasingly common pathologies with a high rate of medical comorbidities [1]. It is essential to be able to safely and expeditiously perform surgical interventions to be able to mobilize these patients as soon as possible to avoid further complications from these injuries [2, 3]. General anesthesia is a reliable method but can have many complications in patients with multiple comorbidities [4, 5]. Neuraxial anesthesia is an excellent alternative to general anesthesia, but complication rates are increased when patients are on anticoagulation which is common for hip fracture patients [6,7]. In addition, neuraxial techniques such as spinal or epidural anesthesia require lateral decubitus or seated positioning that can be extremely difficult with medically frail patients with a hip fracture. 

There is a need for a safe, reproducible anesthetic technique with minimal systemic physiologic impact for medically complex hip fracture patients undergoing minimally invasive, percutaneous surgery. Surgery under local anesthesia only with or without monitored anesthesia sedation has been well described and successful in distal extremity surgery [8,9]. Monitored Anesthesia Care and Soft-Tissue Infiltration of Local Anesthetic (MAC-STILA) has been used at our institution for medically complex patients requiring percutaneous hip fracture fixation to avoid complications associated with general or neuraxial anesthesia [10].

The purpose of this technical guide is to describe the MAC-STILA technique for operative repair of hip fractures using a short cephalomedullary nail (CMN).

## Case presentation

History of present illness

**Figure 1:**
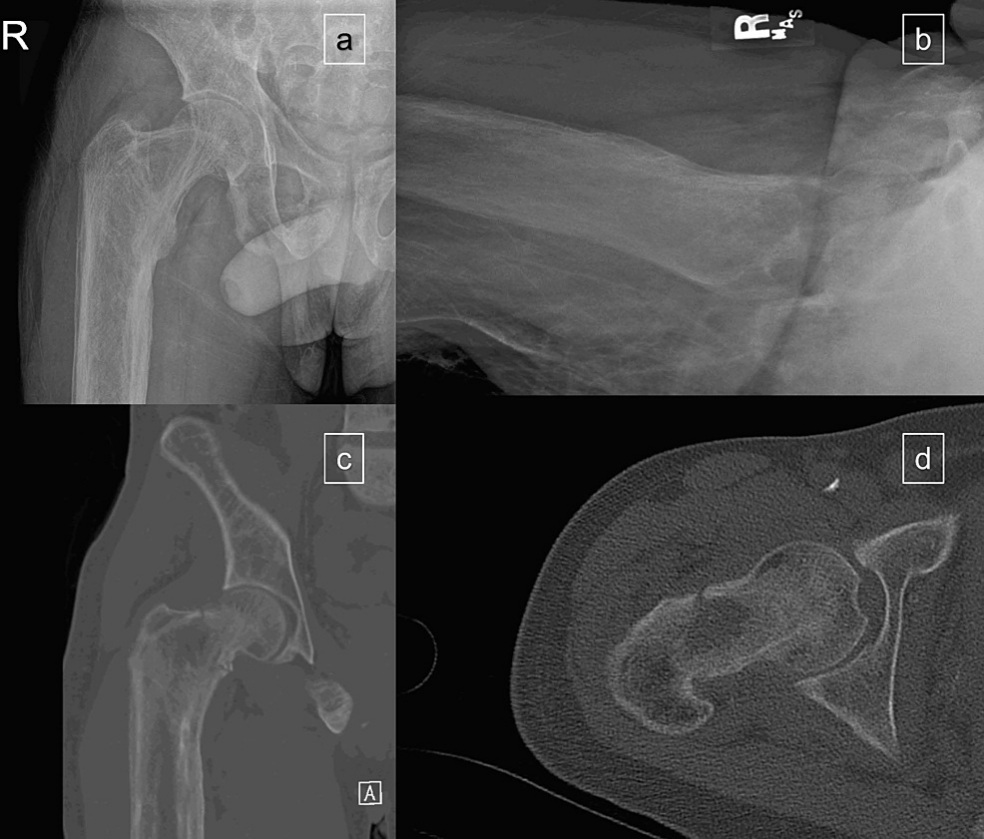
Preoperative radiographs (a and b) and CT scans (c and d) of a right basicervical (OTA/AO 31-B2.1) femoral neck fracture. Note the osseous abnormalities resultant from the patient’s renal osteodystrophy.

The patient is a 32-year-old male with a past medical history of congestive heart failure with reduced ejection fracture (preoperative transthoracic echocardiogram ejection fraction of 40%), end-stage renal disease on hemodialysis with resultant renal osteodystrophy, tricuspid regurgitation and severe pulmonary hypertension who sustained a mechanical trip and fall with a resultant right hip basicervical (OTA/AO 31-B2.1) femoral neck fracture (preoperative radiographs and CT scan in Figure 1). The patient was alert and oriented and neurovascularly intact and had an additional injury of an L4 compression fracture. The patient was evaluated by the medical and cardiology services and deemed to have a revised cardiac risk index class 2 risk with a 6% 30-day risk for myocardial infarction, cardiac arrest, and death, and to be at risk of decompensated heart failure. To minimize the patient’s intraoperative cardiopulmonary event risk, the MAC-STILA anesthetic technique was chosen. The patient was brought to the operating room less than 23 hours from admission.

Surgical technique

In the operating room, vital signs monitors are applied and sedation is administered consistent with monitored anesthesia care (MAC) prior to transferring the patient. Sedative combinations commonly used at our institution include a ketamine/versed/fentanyl or precedex/propofol/fentanyl, but sedative choices should be discussed closely with the anesthesia team.

The patient is then transferred while under MAC from the stretcher to the fracture table. The level of sedation is such that the patient may adequately respond to commands and questions. The operative lower extremity is positioned in a fracture boot. The patient’s contralateral lower extremity is scissored below the level of the operative extremity and secured to the table. Gentle gross traction can be applied at this point to better align the leg.

**Figure 2:**
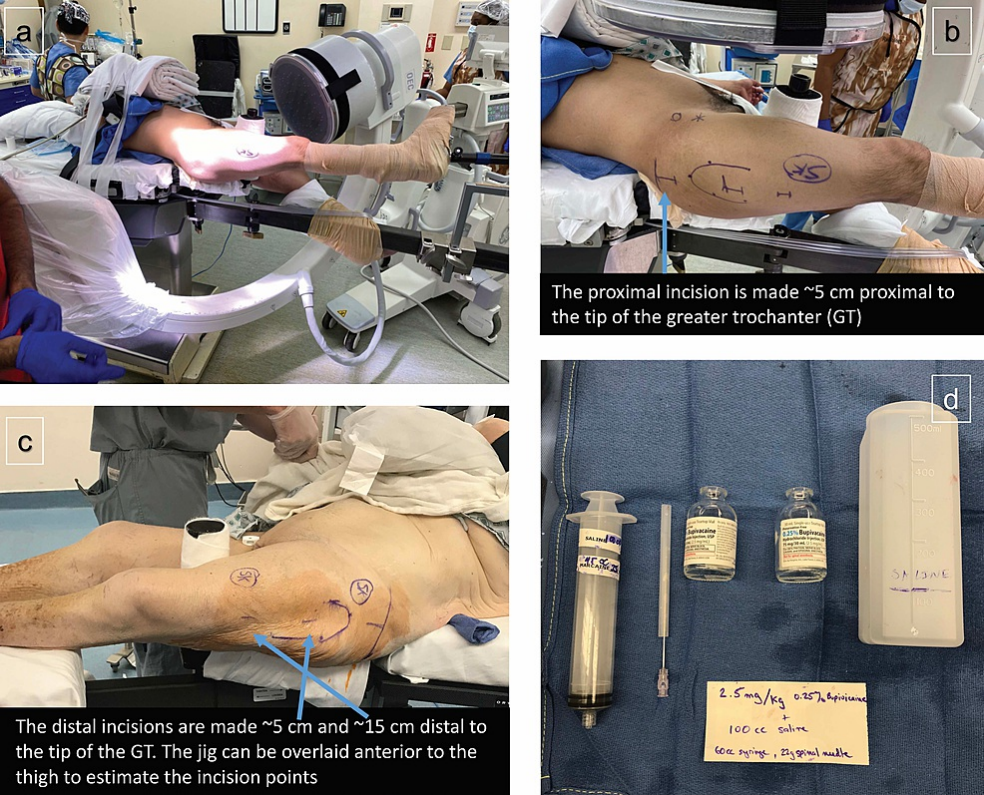
Preoperative preparation for monitored anesthesia care and soft-tissue infiltration of local anesthetic (MAC-STILA) a) Patient positioning for short cephalomedullary nailing (CMN). b) Landmarks marked for short CMN incisions, as well as targets for the STILA corresponding to the nail entry point to the greater trochanter, the head lag screw, and the distal interlock screw. c) Similar landmarks for a different patient for a left hip. d) Supplies required for STILA: 2.5 mg/kg of 0.25% bupivacaine, 22-gauge spinal needle, 50-60 cc syringe, 0.9% normal saline.

The surgical anatomical landmarks (tip of the greater trochanter, vastus ridge) are marked. Next, markings are made for the three planned surgical incisions: 5 cm proximal to the tip of the trochanter (starting point incision), 5 cm distal to the tip of the trochanter (incision for cephalad lag screw), and 15 cm distal to the tip of the trochanter (incision for the targeted locking bolt for the nail) (Figure 2a-2c). These last two measurements correspond with screw openings in the Stryker Gamma3 Short (170 mm and 180 mm) Trochanteric Nail® (Stryker, Kalamazoo, USA) and will vary based on the specific implant used. Next, the lateral aspect of the leg is prepped sterile just for the STILA portion of the procedure. 

Supplies needed for the soft-tissue infiltration with local anesthesia include: one 60 cc syringe, one 20/21/22 gauge spinal needle, 100-cc sterile saline, and 0.25% (2.5 mg/ml) bupivacaine. Appropriate volume of bupivacaine for this procedure is 2.5 mg/kg - 3.0 mg/kg (Figure 2d). For a typical 70 kg patient, this is around 60 cc of 0.25% bupivicaine. This is diluted in 100 cc of sterile saline which is used solely as a volume expander and diffusing agent to allow the anesthetic to better penetrate around the soft tissues. Using the 60 cc syringe and spinal needle, bupivacaine is administered to all surgical sites from dermis, through subcutaneous tissue, muscle, down to periosteum, using fluoroscopy as needed for visualization of the tip of the greater trochanter, vastus ridge, lateral aspect of the femoral shaft, and fracture site. 

**Figure 3:**
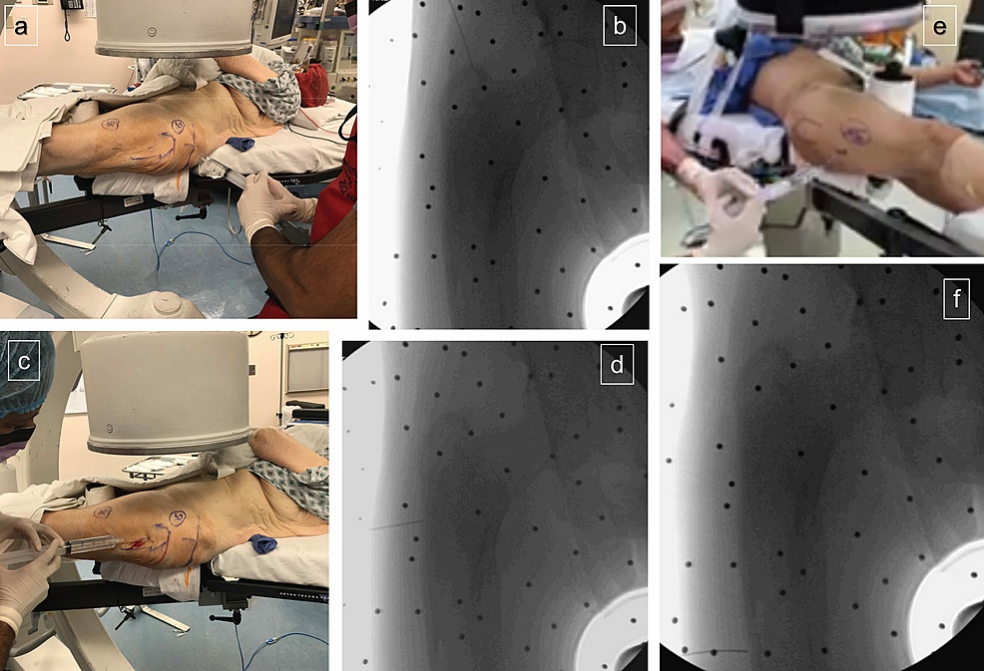
Soft-tissue infiltration of local anesthetic (STILA) procedure a) 50% total volume of bupivacaine solution injected in the proximal nail entry trajectory from the skin down to the tip of the greater trochanter (GT) periosteum. b) Intraoperative fluoroscopy demonstrating spinal needle at GT. c) 30% total volume of bupivacaine solution injected in the lag screw trajectory from the skin down to the vastus ridge. d) Intraoperative fluoroscopy demonstrating spinal needle at the vastus ridge. e) 20% total volume of bupivacaine solution injected in the distal interlock trajectory from the skin down to lateral femur. f) Intraoperative fluoroscopy demonstrating spinal needle at the lateral femoral cortex.

For the start point incision, 50% of the total volume of bupivacaine/saline solution is used as this is the area that is subjected to the most iatrogenic trauma during the procedure. The injection should be directed along the epidermis and dermis, through the subcutaneous fat in a proximal to distal trajectory, through iliotibial band fascia and the gluteus medius muscle and tendon and down to the greater trochanter periosteum. It is important to not end the administration just at the tip of the greater trochanter but to infiltrate as much of the proximal femur as possible. A repetitive back and forth motion with the spinal needle is helpful as is a second injection starting more distal on the skin, along a line from the femoral head to the tip of the greater trochanter, to ensure adequate infiltration of the proximal femur from lateral to medial (Figure 3a-3b).

Next, local anesthetic is administered to the cephalad lag screw incision site. Thirty percent of the total volume of bupivacaine/saline is used and it is injected along the entire incision from the skin to the vastus ridge on the lateral aspect of the femur through subcutaneous fat in a distal to proximal trajectory, through the vastus lateralis fascia and muscle, to the vastus ridge periosteum (Figure 3c-3d). The last 20% of the bupivacaine/saline is injected into the most distal incision site used for the distal locking bolt which anesthetizes the skin, subcutaneous tissue, vastus lateralis muscle, and lateral femur periosteum (Figure 3e-3f).

**Figure 4:**
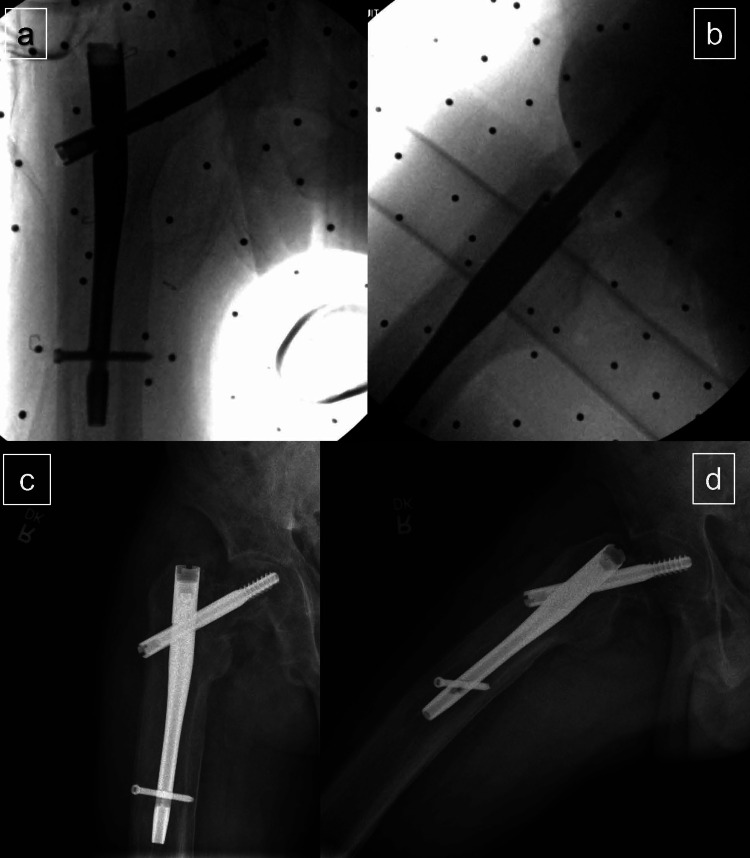
Post-operative fluoroscopy images after short cephalomedullary nailing anteroposterior (AP) (a) and cross-table lateral (b). One-year follow-up AP (c) and cross-table lateral (d) radiographs

The hip fracture is then reduced under fluoroscopy on the fracture table with standard techniques, and the extremity is subsequently prepped and draped for the surgical procedure. Standard repair of the hip fracture using a short CMN is performed (Figure 4). 

Postoperative course

Postoperatively, the patient recovered in the post-anesthesia care unit and was transferred to a monitored room with continuous telemetry and pulse oximetry under the medical service. Standard postoperative analgesia is provided through oral acetaminophen 1000 mg every eight hours scheduled, oral tramadol 50 mg every six hours as needed, and oral oxycodone 5 mg every six hours as needed. It is standard practice at our institution to also provide patients with intravenous ketorolac 15 mg every six hours for the first 24 hours, followed by oral ibuprofen 600 mg every six hours as needed, however, these non-steroidal anti-inflammatory drugs (NSAIDs) were held due to the patient’s renal disease. The postoperative hemoglobin and hematocrit were stable at 9.4 and 28.1, respectively, from pre-operative 11.3 and 34.5, and they were able to ambulate 50 feet with physical therapy and a rolling walker on postoperative day one. No medical complications occurred during his hospital stay, and the patient was ultimately discharged home on postoperative day 10. At the most recent one-year follow-up, the patient was doing well, with minimal operative site pain, and able to ambulate without an assistive device to and from his medical appointments. One-year radiographs are demonstrated in Figure 4c-4d.

## Discussion

Hip fractures occur commonly in the medically complex patient population, yet there is limited literature and recommendations on performing surgical interventions on these patients using options that do not involve general or neuraxial anesthesia. The senior author has published a case-control series comparing the MAC-STILA technique for hip fracture repair to a matched cohort of spinal and general anesthesia patients and demonstrated improved maintenance of intraoperative physiologic parameters with MAC-STILA and similar postoperative pain control compared to spinal anesthesia up to 72 hours postoperatively [10]. This Technical Note article more clearly elucidates the technique for reproducibility at other institutions. To the author’s knowledge, there are no other studies that describe the use of MAC-STILA in medically complex patients with hip fractures in order to expeditiously and safely achieve early surgical fixation and mobilization for these patients.

MAC-STILA is used in our institution for percutaneous fixation of hip fractures with short CMNs or percutaneous cannulated screws. Both procedures have an average surgical time of around 30 minutes, however, given that the half-life of bupivacaine is three hours this technique can certainly be used for procedures that are expected to take longer. Based on the senior authors' anecdotal experience, the points during the procedure when the patient may experience some minimal amount of pain are during the fracture reduction portion of the procedure, and the initial opening reaming of the tip of the greater trochanter. However, when questioned postoperatively patients have no recollection of experiencing any pain. Thus, we do not recommend MAC-STILA as an anesthetic option for displaced reverse obliquity or subtrochanteric fractures, which commonly require more extensive reduction techniques, such as extensive manipulation, percutaneous techniques such as ball spikes or bone hooks, or even open reduction. In addition, these fracture patterns require long CMN, and if an intramedullary reamer is necessary to widen the isthmus of the femoral canal, this may also momentarily increase the pain level beyond tolerable levels to the sedated patient. 

There are still complications to be aware of when performing MAC-STILA in medically complex patients. Systemic local anesthesia toxicity can occur, either from inadvertent intravascular injection or vascular uptake from a large amount of local injected into a localized soft tissue area [11]. Symptoms include but are not limited to perioral numbness, tinnitus, arrythmias, cardiovascular collapse, seizures, and coma. Although there exists a well-described treatment with 20% fat emulsion, patients should be appropriately counseled in the preoperative setting by both anesthesiologists and orthopedic surgeons [12]. While MAC-STILA is used in our institution for procedures such as short CMN and percutaneous cannulated screws, we do not recommend this anesthetic option for procedures that consistently require beyond 30 minutes of incision to closure time, such as total hip arthroplasty or hemiarthroplasty for the treatment of displaced femoral neck fractures.

## Conclusions

The purpose of describing this technique is to provide orthopedic surgeons and anesthesiologists with another option beyond general anesthesia and neuraxial anesthesia for hip fracture care in medically complex patients. By avoiding the systemic effects of general anesthesia, we believe this can reduce cardiovascular complications, respiratory complications, and postoperative delirium in the postoperative period following surgical fixation of hip fractures. In addition, by providing another anesthetic option to avoid the contraindication to neuraxial anesthesia in the setting of anticoagulation, MAC-STILA can prevent delays to operative intervention for hip fractures without impacting postoperative recovery and mobilization. We hope this report provides a template to guide future surgeons when performing MAC-STILA for short CMN.

## References

[1] Zuckerman JD (1996). Hip fracture. N Engl J Med.

[2] Hung WW, Egol KA, Zuckerman JD, Siu AL (2012). Hip fracture management: tailoring care for the older patient. JAMA.

[3] Kenyon-Smith T, Nguyen E, Oberai T, Jarsma R (2019). Early mobilization post-hip fracture surgery. Geriatr Orthop Surg Rehabil.

[4] Tzimas P, Samara E, Petrou A, Korompilias A, Chalkias A, Papadopoulos G (2018). The influence of anesthetic techniques on postoperative cognitive function in elderly patients undergoing hip fracture surgery: general vs spinal anesthesia. Injury.

[5] Short TG, Campbell D, Frampton C (2019). Anaesthetic depth and complications after major surgery: an international, randomised controlled trial. Lancet.

[6] Memtsoudis SG, Sun X, Chiu YL (2013). Perioperative comparative effectiveness of anesthetic technique in orthopedic patients. Anesthesiology.

[7] Guay J, Parker MJ, Gajendragadkar PR, Kopp S (2016). Anaesthesia for hip fracture surgery in adults. Cochrane Database Syst Rev.

[8] Wright J, MacNeill AL, Mayich DJ (2019). A prospective comparison of wide-awake local anesthesia and general anesthesia for forefoot surgery. Foot Ankle Surg.

[9] Evangelista TM, Pua JH, Evangelista-Huber MT (2019). Wide-Awake Local Anesthesia No Tourniquet (WALANT) versus local or intravenous regional anesthesia with tourniquet in atraumatic hand cases in orthopedics: a systematic review and meta-analysis. J Hand Surg Asian Pac Vol.

[10] Konda SR, Ranson RA, Dedhia N, Tong Y, Saint-Cyrus E, Ganta A, Egol KA (2021). Monitored anesthesia care and soft-tissue infiltration with local anesthesia: an anesthetic option for high-risk patients with hip fractures. J Orthop Trauma.

[11] Neal JM, Barrington MJ, Fettiplace MR (2018). The third American Society of Regional Anesthesia and Pain Medicine practice advisory on local anesthetic systemic toxicity: executive summary 2017. Reg Anesth Pain Med.

[12] Becker DE, Reed KL (2006). Essentials of local anesthetic pharmacology. Anesth Prog.

